# Neurodegeneration in a *Drosophila* Model for the Function of TMCC2, an Amyloid Protein Precursor-Interacting and Apolipoprotein E-Binding Protein

**DOI:** 10.1371/journal.pone.0055810

**Published:** 2013-02-07

**Authors:** Paul C. R. Hopkins

**Affiliations:** Institute of Psychiatry and MRC Centre for Developmental Neurobiology, Kings College London, London, United Kingdom; Oregon Health and Science University, United States of America

## Abstract

We previously identified TMCC2 as a protein that interacted differentially with normal versus Alzheimer's disease-risk forms of both apolipoprotein E (apoE) and the amyloid protein precursor (APP). We hypothesized that disrupted function of TMCC2 would affect neurodegeneration. To test this hypothesis, we investigated the *Drosophila* orthologue of TMCC2, that we have named Dementin. We showed that Dementin interacts genetically both with human APP and its *Drosophila* orthologue, the APP-like protein (APPL). Ectopic expression of Dementin in *Drosophila* rescued developmental and behavioral defects caused by expression of human APP. Both a hypomorphic lethal mutation in the *dementin* gene (*dmtn^1^*) and RNAi for Dementin caused the accumulation of fragments derived from APPL. We found that Dementin was required for normal development of the brain, and that glial Dementin was required for development of the *Drosophila* medulla neuropil. Expression of wild-type Dementin in either the neurons or glia of *dmtn^1^* flies rescued developmental lethality. Adult *dmtn^1^* flies rescued by expression of wild-type Dementin in glia, i.e. whose neurons expressed only *dmtn^1^*, showed pathological features resembling early onset Alzheimer's disease, accumulation of abnormal APPL metabolites, synaptic pathology, mis-localized microtubule-binding proteins, neurodegeneration, and early death.

## Introduction

Pathologically, Alzheimer's disease (AD) is defined by amyloid plaques, increased levels of mislocalized tau, and is accompanied by glia-mediated inflammation. This process leads to loss of synaptic markers and neuronal death, with the consequence that in advanced AD the brain is atrophied, leading to severe cognitive and behavioral deficits.

Genetic studies provide the strongest evidence for causative agents in AD, these show that mutations in presenilin 1, presenilin 2 or the amyloid protein precursor (APP) cause early-onset AD in an autosomal dominant manner, as does duplication of the gene for APP [1]–[4]. Although mutations in APP or presenilin are rare, late- and early-onset AD are pathologically similar, and late-onset AD is strongly linked to polymorphisms of the gene for apolipoprotein E (apoE) [5]. Meta-analysis of the numerous studies confirming the role of apoE in AD show that the lifetime risk of AD in people homozygous for the APOE*4 isoform of apoE may be as high as 64% whereas in people homozygous for the APOE*2 isoform it may be as low as 0.1% [6].

It is well established that APP and presenilin interact directly, presenilin modifies several aspects of APP metabolism, including trafficking and proteolysis [7], [8]. Proposed functions for APP include roles in synaptogenesis, regulation of gene expression and lipid metabolism [9]. AD-linked mutations in presenilin are found throughout the molecule, and do not consistently alter proteolysis of APP in vitro. However, most AD-causing mutations in APP are restricted to the C-terminal region that interacts with presenilin, and although there are over 30 different proteins known to interact with presenilin [7], to date, APP is the only one found mutated in AD. Thus, a modified interaction between presenilin and APP likely lies at the heart of AD-specific neurodegenerative processes.

A role for apoE in the metabolism of APP, or the presenilin-dependant proteolytic product, Aβ, was revealed when apoE-null mice expressing human APP that carries the “Swedish” mutation (APPswe; K595M/N596L) had a reduced development of fibrillar amyloid plaques when compared to wild-type mice [10], [11]. Furthermore, APPswe mice expressing apoE4 produced higher levels of soluble Aβ and developed fibrillar amyloid plaques at an earlier time point than those expressing apoE3 [12], [13]. ApoE may further have distinct roles depending on whether it is expressed in neurons or glia, as the cellular origin of apoE differentially modifies amyloid pathology in mice, as well as responses to excitotoxicity [14], [15].

We recently identified TMCC2 as a protein that interacted differentially with the AD-risk versus normal forms of both apoE and APP [16]. To test the hypothesis that TMCC2 might play a role in neurodegeneration we examined the *Drosophila* member of this protein family, which we have named Dementin in view of the vital role it plays in brain development. We found that Dementin interacted genetically both with human APP and it's *Drosophila* orthologue, the APP-like protein (APPL). We further showed that adult flies whose neurons expressed only mutated Dementin had AD-like pathology, neurodegeneration and shortened lifespans. Flies mutated for Dementin thus emulated pathological features of early onset AD.

## Results

**Figure 1:**
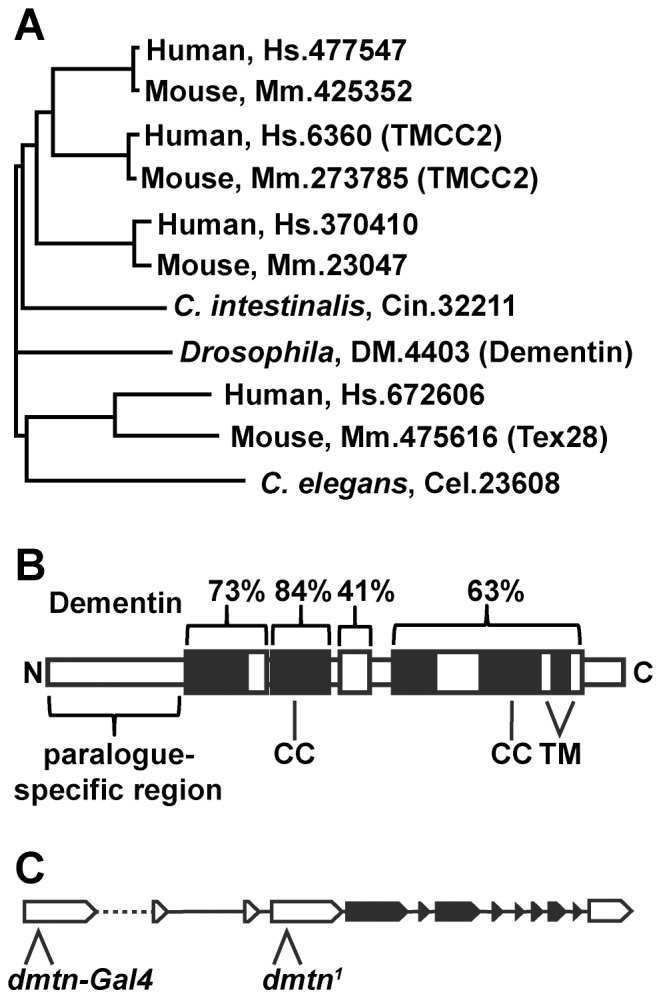
The Dementin Family of proteins. Dendrogram (A) and schematic (B) showing the sequence similarity and structural organization of Dementin and related proteins and their Unigene codes. “CC” represents domains predicted to form coiled-coil structures, and “TM” represents regions predicted to form transmembrane domains. The percent similarity of the domains conserved between TMCC2 and Dementin are indicated by brackets. See Fig. S1 for alignments. (C) Structure of the *Drosophila dementin* gene from flybase.org showing the locations of the enhancer trap (*dmtn-Gal4*) and the lethal mutation (*dmtn^1^*) used in this study; coding exons are in filled boxes and non-coding exons in open boxes, the intron indicated by a dashed line is not to scale.

Dementin (CG1021, Unigene code Dm.4403) is a member of a family of highly conserved proteins with four orthologues in each of the human and mouse genomes and one in the *Drosophila* genome (Table 1, Fig. 1A). Protein sequence alignment showed that *Drosophila* Dementin is 40 to 80% similar to human TMCC2 in the conserved region located within the C-terminal ∼400 amino acids (Fig. 1B; Fig. S1). The N-terminus of this protein family is highly variable between paralogues, yet almost completely conserved between the human and mouse homologues, and therefore likely contributes significantly to differential activity by paralogues, possibly as a regulatory domain.

**Table 1:** Identities of *dementin*-related genes in human, mouse, *D. melanogaster, C. elegans and C. intestinalis*.

Unigene codes and published synonyms of *dementin* orthologues	Chromosomal location
Human, Hs.477547	3q21.3
Human, Hs.6360 (TMCC2)	1q32.1
Human, Hs.370410	12q22
Human, Hs.672606 (Tex28)	Xq28
Mouse, Mm.425352	6qE3
Mouse, Mm.273785 (TMCC2)	1qE4
Mouse, Mm.23047	10qC2
Mouse, Mm.475616 (Tex28)	XqA7.3
*Drosophila*, Dm.4403 (Dementin)	83E5-83E6
*C. elegans*, Cel.23608	X
*C. intestinalis*, Cin.32211	1p

### Expression pattern of Dementin in the central nervous system

**Figure 2:**
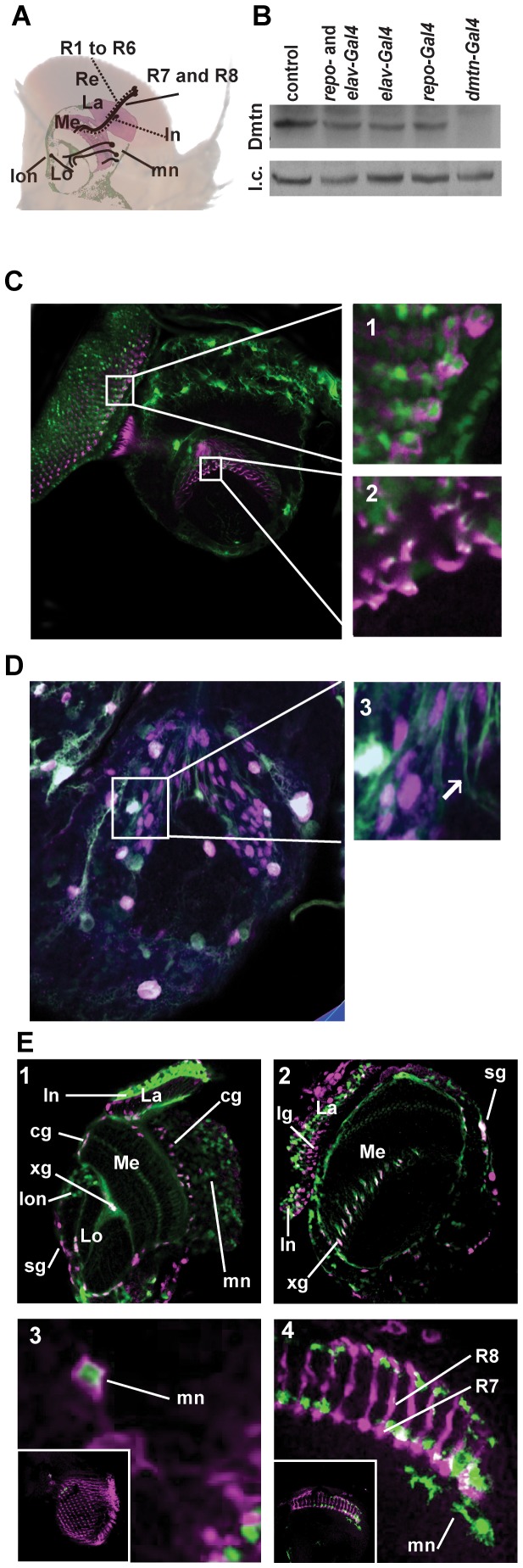
Expression pattern of Dementin in the brain. (A) Schematic of the adult optic lobe. (B) Immunoblot of preparations from the heads of young adult flies which have RNAi-mediated suppression of Dementin in both glia and neurons (*repo- and elav-Gal4*), glia alone (*repo-Gal4*), neurons alone (*elav-Gal4*), or using *dmtn*-Gal4. Each lane was loaded with the equivalent of 3.5 heads prepared from pools of 20–30 heads each. (C and D) Expression of *dmtn-Gal4* driven GFPnls (green) in the optic lobes of white pupae. (C) GFP was detected in the cell body (inset 1) and axons (inset 2) of retinal neurons detected by antibody mAb24b10 against Chaoptin (magenta). GFP was also detected in retinal axons in brains stained for Repo (inset 3, arrowhead). GFP co-localized with the glial transcription factor Repo detected using antibody 8D12 (magenta). See Figs. S4A and S4B for sections of the optic lobes of larvae, animated in movies S1 and S2. (E and F) Expression of *dmtn-Gal4* in the adult optic lobe. (E) Detection of GFPnls (green) in glia and neurons of the adult optic lobe in a homozygous *dmtn-Gal4* fly stained with anti-Repo (magenta). Panel 1, oblique optical section; panel 2, vertical optical section. Panels 3 and 4, examples of *dmtn-Gal4*-driven cell-surface targeted mCD8::GFP (green) using MARCM in neurons of adult optic lobes stained with anti-Chaoptin (magenta). La, lamina; Lo, lobula complex; Me, medulla; Re, retina; cg, cortex glia; lg, lamina glia; sg, surface glia; xg, chiasm glia; ln, lamina neurons, lon lobula neurons; mn, medulla neurons. See Fig. S2C for an image of the adult central brain.

We investigated the functions of Dementin within the *Drosophila* optic lobe, as it is well studied and since comprises a large fraction of the *Drosophila* brain. The *Drosophila* visual system is composed of the retina and three neuropils, the lamina, medulla and lobula complex [17] (Fig. 2A). The ∼800 ommatidia of the *Drosophila* compound eye each contain 8 photoreceptor neurons (R1–R8) that project into the brain where they make a visual map. Neurons R1 to R6, specialized for the detection of moving stimuli, terminate in the lamina, and the information they convey is relayed to the medulla by lamina neurons. Neurons R7 and R8, specialized for color and polarized light detection, pass through the lamina and terminate in discrete layers within the medulla. The medulla, where most visual information processing occurs, comprises approximately 40,000 neurons, which terminate either within the medulla, or pass through it and terminate in the lobula [18].

Dementin is widely expressed in glia and in subsets of neurons of the optic lobe, both dynamically during development and in adults, as determined using a homozygous viable enhancer trap in the *dementin* locus (*dmtn-Gal4*; *P{GawB}CG1021^NP6590^*; Fig. 1C), and through the impact of Dementin expression shown below. Production of Dementin was partly suppressed by either neuron- or glia-specific RNAi, using the pan-neural driver *elav-Gal4*, and the pan-glial driver, *repo-Gal4*, both separately and together, and further suppressed by RNAi driven either with the *dmtn-Gal4* (Fig. 2B), or *tubulin-Gal4* drivers (Fig. S2A), demonstrating the effectiveness of the fly stock used to cause Dementin-targeted RNAi, and further indicating that non-CNS cells in the head also express Dementin. *dmtn-Gal4* activity was transiently detected in the retinal axons of prepupae stained with mAb24b10, an antibody to Chaoptin, a leucine-rich repeat cell adhesion molecule; as the GFP signal partially co-localizes with the chaoptin signal *dmtn-Gal4* is active in photoreceptor neurons, rather than adjacent glial cells (Fig. 2C, inset 2). Expression was also shown in surface and cortex glia by co-localization of nuclear-targeted GFP with antibody 8D12 to Repo, a glia-specific transcription factor (Fig. 2D). In contrast to prepupae, still and animated images produced from confocal optical sections of the optic lobes of third instar larvae expressing nuclear-targeted GFP under control of *dmtn-Gal4* and stained either with mAb24b10 to chaoptin or 8D12 to repo (Figs. S4A and B, and movies S1 and S2) demonstrate expression of *dmtn-Gal4* in glia and neurons of third instar wandering larvae, but not in photoreceptor neurons, indicating that the expression of *dmtn-Gal4* in these cells is dynamically regulated in retinal neurons during development. In adults, widespread expression in the optic lobe and central brain was observed (Fig. S2B and C). As in prepupae, expression of Dementin was found in surface glia, chiasm and cortex glia, as well as in lamina, medulla and lobula neurons (Fig. 2E), but notably not in lamina glia. Fig. 2E, panel 1, shows an oblique section of the optic lobe, Fig. S2B a horizontal section, and Fig. 2E, panel 2, a vertical section. The Dementin expression pattern was also investigated using mosaic analysis (MARCM) in conjunction with *dmtn-Gal4*. This showed expression in subsets of adult medulla neurons (Fig. 2E panel 4), including mAb24b10-positive medulla neurons (Fig. 2E, panel 3), as well as in chiasm glia and projections to the lobula (not shown).

### Dementin interacts genetically with APP and APPL

**Figure 3:**
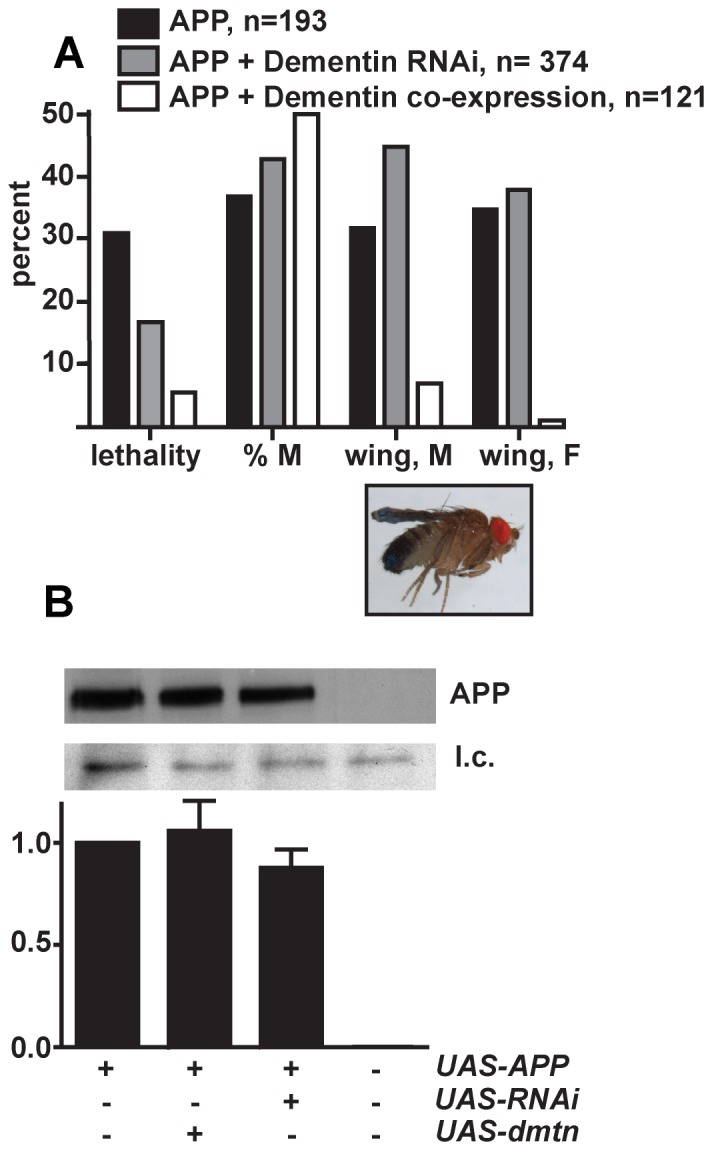
Genetic interaction of Dementin with human APP. (A) eclosion rates and wing expansion phenotypes. The image is of a fly expressing human APP in neurons, showing unexpanded wings. % M, percent of flies eclosing that were male. (B) Immunoblot and densitometric quantification prepared from the heads of male flies expressing human APP (*UAS-APP*), which also have RNAi for Dementin (*UAS-RNAi*), or which ectopically express Dementin (*UAS-dmtn*) driven by *elav-Gal4*. Each sample was prepared from 30 heads and the equivalent of 3 heads was loaded per lane. Replicate immunoblots are shown in Fig. S2G. Densitometry results are expressed as a proportion of the result obtained from flies only expressing APP, corrected by loading control. APP was detected with antibody A5137 targeted against the C-terminus of APP. l.c., loading control. Data are from 10 to 12 independent crosses.

**Figure 4:**
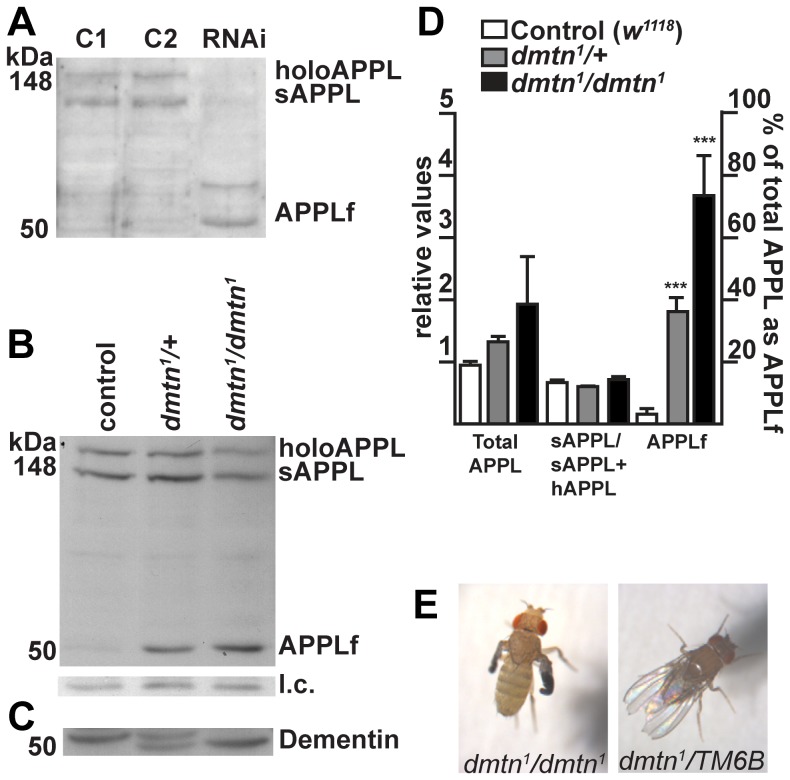
Alteration of APPL metabolism both by RNAi for Dementin and by mutation of *dementin*. (A) Western blot of heads from 10-day-old flies with RNAi for Dementin driven in neurons shows the appearance of a novel ∼50 kDa APPL-derived C-terminal fragment (APPLf). c1 and c2, control parent lines: U*AS-Dcr2;+;UAS-RNAi/TM6B* and *elav-Gal4;+;+*; RNAi, progeny with RNAi for Dementin: *Dcr2/elav-Gal4; +; UAS-RNAi/+*. Each lane was loaded with equivalent of 3 heads per lane from preparations made with 20 heads each. (B) Western blot of heads from *dmtn^1^* flies: *dmtn^1^* also induced production of the ∼50 kDa APPLf. Each lane is loaded with the equivalent of 4 heads from preparations made from 20–40 fly heads collected on the day of their eclosion. All flies were 9 generations isogenic. APPL was detected in all blots using antibody dR-14, the APPLf was also detected in each case with antibody dC-21 (not shown). See Fig. S2E for replicates. (C) Western blot for Dementin from the same samples showing a truncated Dementin peptide. l.c., loading control. (D) Densitometric quantification of (B) showing that *dmtn^1^* does not affect total levels of APPL or the ratio of holoAPPL to sAPPL, but does induce the production of APPLf (p<0.0001). Values for total APPL and the ratio of sAPPL to sAPPL+hAPPL are expressed as relative to that obtained with control *w^1118^* flies (left-hand scale); the percent of total APPL that was APPLf is shown on the right-hand scale. (E) Wings of homozygous *dmtn^1^* flies (left), but not heterozygous controls (right, *dmtn^1^/TM3 Ser*) fail to expand after eclosion. Both images were captured 3 h after eclosion. Error bars represent the SEM.

A human orthologue of Dementin, TMCC2, interacts with APP [16]. We found that *dementin* interacts genetically both with human APP and with its *Drosophila* orthologue, APPL. Neuronal expression of human APP in *Drosophila* during development is semi-lethal, affecting males more severely than females; it also causes a failure of wings to expand after eclosion [19], [20], a behavioural phenotype controlled by the CNS [21]. Similar effects are caused by expression of APPL that lacks a portion of its extracellular domain (APPLsd [22]). We found that co-expression of Dementin with APP in neurons protected against APP-induced developmental lethality and against wing-expansion defects; while knockdown of Dementin expression in neurons using RNAi rescued in part developmental lethality, it exacerbated the wing expansion phenotype (Fig. 3A). These effects were found to be independent of variations in the level of APP expression (Fig. 3B, Fig. S2G). Neither global nor neuron-specific ectopic expression of Dementin revealed overt phenotypes (not shown). Since the *Drosophila* orthologue of APP, APPL, would be the physiological functional partner for Dementin, rather than APP, we investigated the effect of Dementin on APPL metabolism. In independently performed experiments, we found that either neuronal RNAi for Dementin or mutated dementin alleles modified the metabolism of APPL. 10-day-old adult flies with RNAi for Dementin in neurons showed both a loss of full-length APPL and the appearance of a ∼50 kDa C-terminal fragments (APPLf; Fig. 4A, S2D). Two dementin alleles, the piggyBac insertions f07621 and e01970 were also found to modify APPL metabolism and produced a similar ∼50 kDa APPLf (Fig. S2D).

Dementin is expressed in several larval and adult tissues in addition to the CNS (observations from *dmtn-Gal4* flies not shown, and data at www.flybase.org). Therefore, to provide an alternative to CNS-specific RNAi to investigate the role of Dementin in the brain, we examined the effect of several *dmtn* alleles on CNS development. We found *dmtn* alleles that were both developmentally lethal and had severe adult brain phenotypes, including the piggyBac insertions f07621, c01010, c01120, c06041, c06042 and e01970. We selected e01970 (a piggyBac insertion in a 5′ untranslated exon, hereafter called *dmtn^1^*, Fig. 1C) for further study since it was rescued both by neuron-specific and by glia-specific expression of wild-type Dementin (see below). *dmtn^1^* flies produce a truncated Dementin protein (Fig. 4C), as detected using an antibody to a Dementin-derived peptide. This insertion therefore likely causes incorrect splicing of the mRNA or incorrect initiation of translation, causing partial loss of the orthologue-specific N-terminal putative regulatory region of this protein (Fig. 1B, Fig. S1).

*dmtn^1^* flies showed abnormal metabolism of APPL, and produced an APPLf similar to that produced in flies with neuron-specific RNAi for Dementin, as detected by two separate antibodies to APPL (Fig. 4B). In contrast to the two novel APPLf bands seen following RNAi for Dementin, *dmtn^1^* flies produced a single novel APPLf. As the truncated Dmtn^1^ peptide still retains the evolutionarily conserved region of this protein family, this difference may indicate that Dmtn^1^ retains a partial interaction with APPL. Escaper homozygous *dmtn^1^* flies also phenocopied flies with neuronal expression of human APP or APPLsd with respect to wing expansion: 85% of escaper *dmtn^1^* flies (30 out of 35) showed wing expansion defects (Fig. 4E); thus production of this APPLf may in part functionally recapitulate the effect of APPLsd [22] expression. We quantified the levels of APPL and its fragments in *dmtn^1^* and control flies by densitometry of western blots shown in Fig. 4B and Fig. S2E. ANOVA followed by Tukey's multiple comparison test showed that homozygosity for the *dmtn^1^* allele was associated with a trend towards an increase in total levels of APPL (p = 0.042), however *dmtn^1^* homozygotes had significantly smaller brains than *dmtn^1^* heterozygotes and controls, and were not viable (see below), indicating numerous likely indirect effects of Dementin deficiency. However, *dmtn^1^* heterozygotes also showed a similar phenotype with respect to APPL, and no significant difference in the total levels of APPL was found between controls and *dmtn^1^* heterozygotes, which have normal neuroanatomy and are viable. The levels of APPLf were significantly increased in *dmtn^1^* heterozygotes compared to controls (p = 0.0001), and in *dmtn^1^* homozygotes compared to either controls (p = 0.0001) or *dmtn^1^* heterozygotes (p = 0.001) (Fig. 4D). However, we found no significant effect of *dmtn^1^* on the cleavage of APPL by *Drosophila* α-secretases in the circumstances tested, as assessed by calculating the fraction of normal APPL that was found as sAPPL. Thus, the effects of heterozygosity for *dmtn^1^* on APPL metabolism are independent of the effect of homozygosity for *dmtn^1^* on viability and brain development. ∼50 kDa fragments of human APP were not detected when human APP was expressed either in flies with RNAi for Dementin in neurons or in a *dmtn^1^* background (data not shown).

### Dementin is required for normal brain development

**Figure 5:**
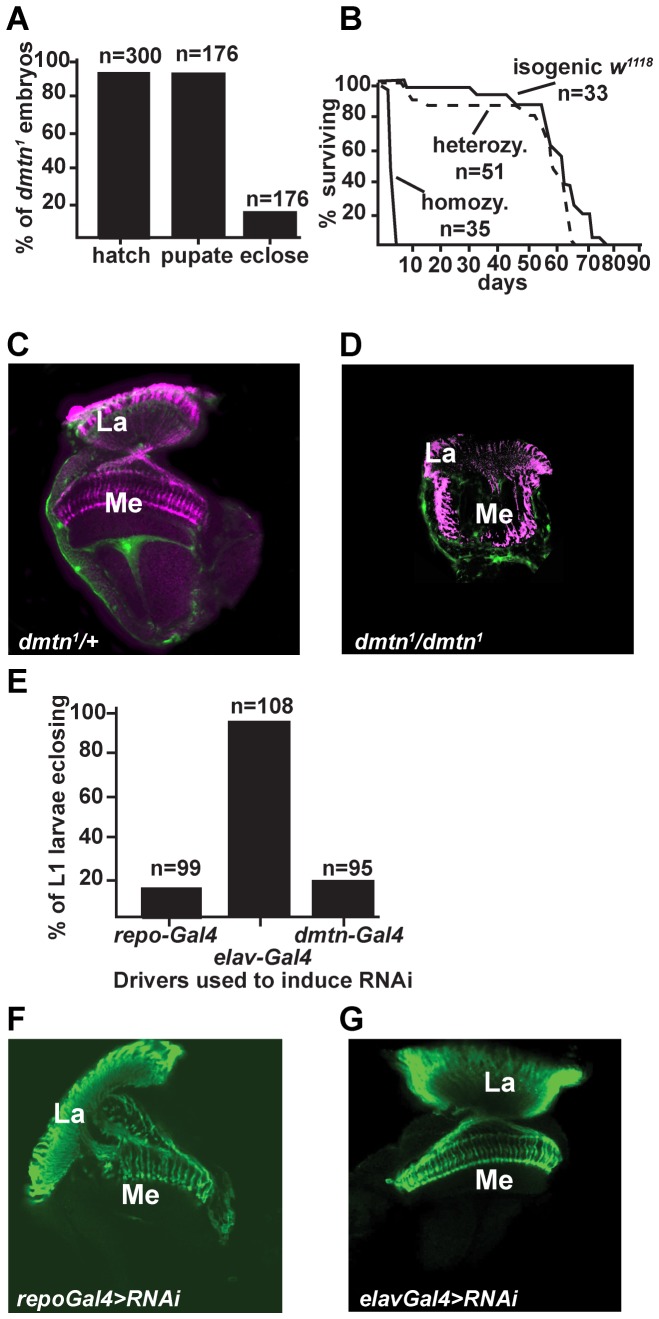
The effect of Dementin on survival and optic lobe development. (A) *dmtn^1^* is a pupal lethal allele. Bars represent the percentage of *dmtn^1^* flies that remain alive at various stages of development. (B) The survival rate of adult *dmtn^1^* flies and controls; all flies were 9 generations or more isogenic. (C and D) *dmtn^1^* flies have an underdeveloped medulla. Retinal axons projections into the optic lobe of heterozygous (C) and homozygous (D, n>26) *dmtn^1^* flies expressing GFPnls under control of *repo-Gal4* (green) were stained with mAb24b10 against Chaoptin (magenta). (E) RNAi for Dementin driven in glia with *repo-Gal4* and with *dmtn-Gal4*, but not in neurons with *elav-Gal4* is developmentally lethal. For a Western blot of extracts prepared from the heads of these flies see Figure 2B. (F and G) Optic lobes stained with antibody mAb24b10 for retinal axons show that RNAi for Dementin driven in glia with *repo-Gal4* (F, n = 8), but not in neurons with *elav-Gal4* (G, n = 12), recapitulates the medulla defects seen in *dmtn^1^* flies. La, lamina; Me, medulla.

We next investigated if Dementin would have an important role either in brain development or in neurodegeneration. Most homozygous *dmtn^1^* flies died during metamorphosis (Fig. 5A); adult escapers died within a few days, whereas heterozygous flies had a normal lifespan (Fig. 5B). Examination of the brains of newly eclosed *dmtn^1^* flies showed that the optic lobes of homozygous but not heterozygous *dmtn^1^* flies were severely reduced in size, and missing much of the medulla neuropil (Fig. 5C and D).

As Dementin is expressed in both neurons and glia, we tested the effect of either neuron- or glia-specific RNAi for Dementin on development and the optic lobe phenotype. An important role for glial Dementin was found. Glia-specific ablation of Dementin production by RNAi recapitulated both the *dmtn^1^* developmental lethality and optic lobe phenotypes (Fig. 5E, F and G). Similar to *dmtn^1^* flies, the lamina of flies with RNAi for Dementin driven specifically in glia was less affected than the medulla, and these flies did not display a readily apparent rough eye phenotype (not shown). RNAi for Dementin driven by *dmtn-Gal4* caused a similar developmental lethality as glia-specific RNAi driven by *repo-Gal4*: 90.4% of flies with RNAi failed to eclose (n = 95, from 3 independent crosses). However, the optic lobe phenotype in flies with *dmtn-Gal4*-driven RNAi for Dementin RNAi flies was weak (data not shown), possibly due either to the dynamic nature of the *dmtn-Gal4* expression pattern in the optic lobe during development and/or the fact that simultaneous expression of Dementin and initiation of the processes that lead to RNAi-mediated degradation of the mRNA may allow sufficient Dementin protein to be produced before the mRNA is degraded.

**Figure 6:**
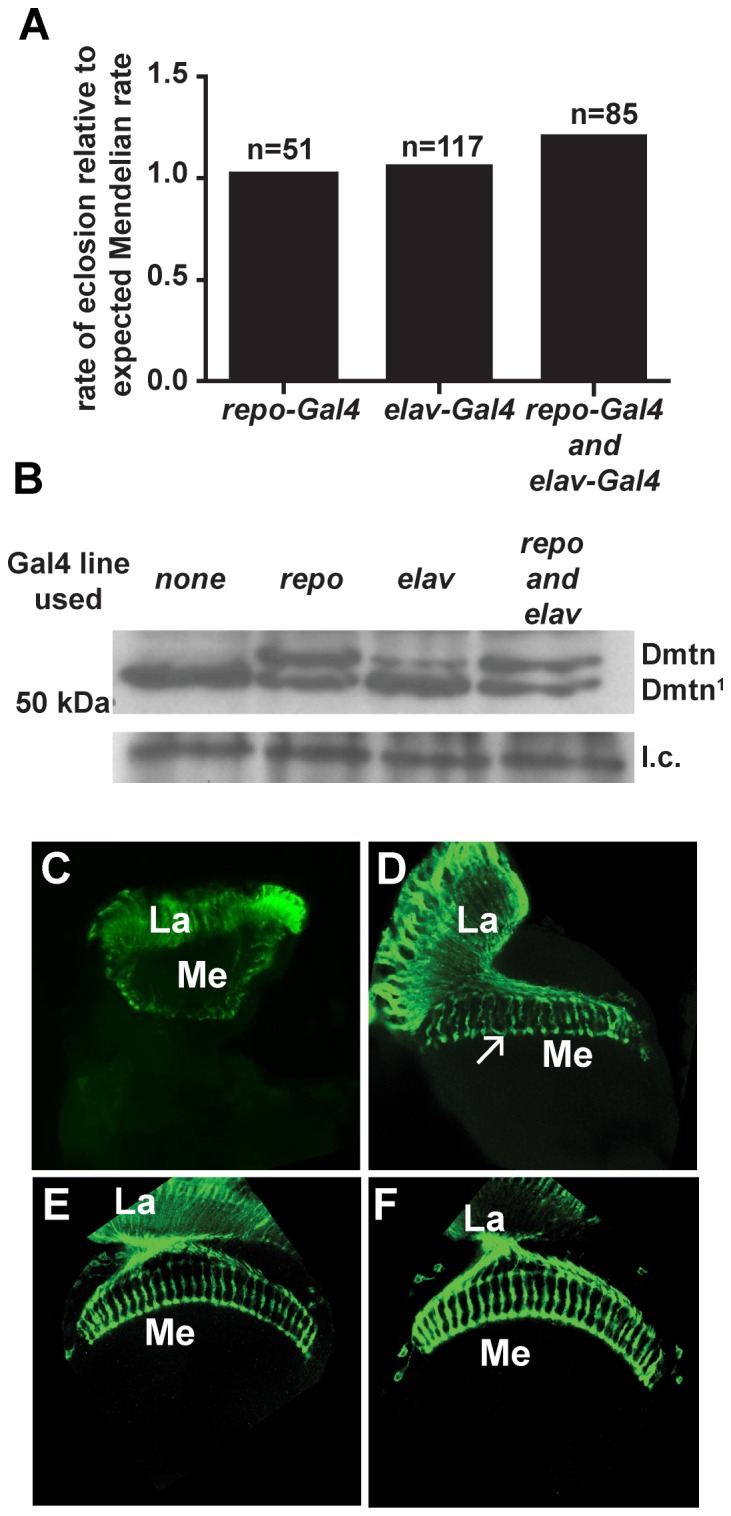
Rescue of *dmtn^1^* flies by wild-type Dementin. The optic lobe defect of *dmtn^1^* flies is fully rescued by expression of Dementin in neurons, and partly by expression in glia. (A) Eclosion rate relative to the expected Mendelian ratio of homozygous *dmtn^1^* flies where wild-type Dementin expression was driven from a *UAS-dmtn* construct in either glia (*repo-Gal4*), neurons (*elav-Gal4*), or in both. (B) Western blot showing expression of Dementin in the same flies. Each lane was loaded with the equivalent of 3 heads prepared from pools of 20–30 heads. l.c., loading control. Corresponding data for Dementin in *w^1118^* and *dmtn^1^* flies is shown in Fig. 4C. (C–F) Representative images of retinal axon projections stained with mAb24b10 in the optic lobe of homozygous *dmtn^1^* flies not rescued by wild-type Dementin (C, n>26), rescued by wild-type dementin specifically in glia (D, n = 11), neurons (E, n = 20) or in both glia and neurons (F, n = 12). The arrow indicates incorrectly targeted retinal axons in *dmtn^1^* flies rescued only in glia. La, lamina; Me, medulla.

A significant role for neuronal Dementin in development was uncovered in rescue experiments. We found that the developmental lethality in *dmtn^1^* flies was rescued by expression of wild-type Dementin specifically in either neurons or glia, or in both, using *elav-Gal4* and *repo-Gal4* drivers (Fig. 6A and B). The optic lobes of all *dmtn^1^* flies rescued by expression of wild-type Dementin in neurons only were normal in appearance (Fig. 6E), as were those of all flies rescued by specific expression in both neurons and glia (Fig. 6F). We found that while expression of wild-type dementin in the glia of *dmtn^1^* flies completely rescued developmental lethality (Fig. 6A), the optic lobes of all *dmtn^1^* flies rescued by expression of wild-type Dementin in glia only were less developed than those of *dmtn^1^* flies rescued by expression of Dementin in neurons, as shown by a wider angle between the lamina and medulla neuropils, due to partial rotation of the medulla neuropil, and by aberrant targeting of retinal axons (Fig. 6D).

### The dmtn^1^ allele can cause neurodegeneration

We next found that mutation of Dementin could cause neurodegeneration. We fixed and embedded in paraffin 1-day-old and 10-day-old *dmtn^1^* flies rescued by expression of wild type Dementin in either neurons or glia. To ensure that the observations made in *dmtn^1^* flies rescued by expression of Dementin in glia were not caused by a second site mutation on the recombined third chromosome containing both *dmtn^1^* and *repo-Gal4*, *dmtn^1^* flies rescued by wild-type Dementin in both neurons and glia were prepared in a similar fashion both 1 day after eclosion and 30 days later. We analyzed our flies for Futsch, the *Drosophila* orthologue of mammalian microtubule associated protein 1B (MAP1B), and which like human tau (but unlike *Drosophila* tau [23]) is normally restricted to the axonal compartment [24]. We also stained sections for Bruchpilot, a component of the presynaptic active zone that contains domains with homology to both vertebrate presynaptic active zone proteins ELKS and CAST [25].

**Figure 7:**
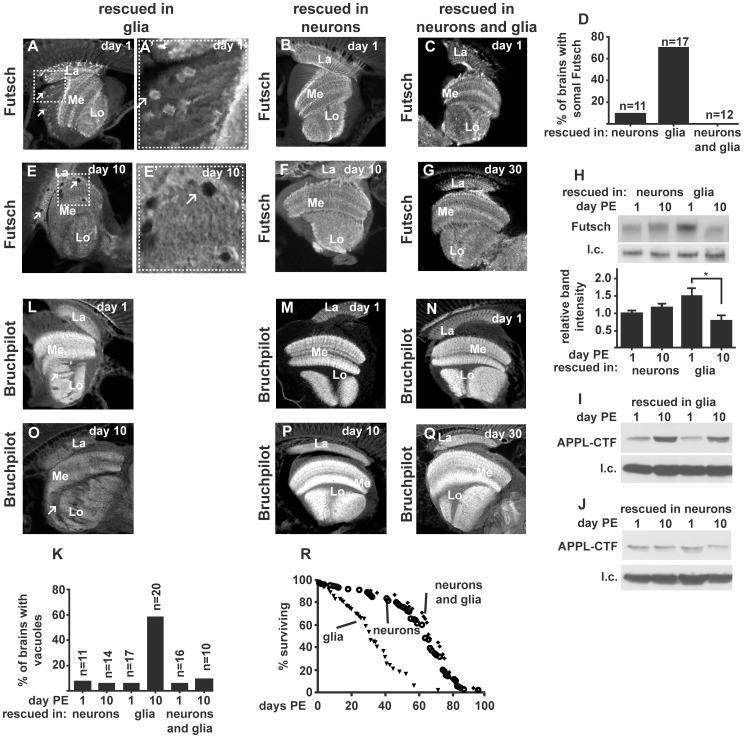
AD-like pathology and neurodegeneration in rescued *dmtn^1^* flies. *dmtn^1^* flies whose neurons express only Dmtn^1^ showed mislocalization of Futsch/MAP1B (A, enlarged in A′), whereas flies whose glia express only Dmtn^1^ (B) did not; neither did *dmtn^1^* flies expressing wild-type Dementin in both neurons and glia (C). Quantification of these data is shown in panel D. All flies whose neurons express only Dmtn^1^ (L) showed patchy staining for Bruchpilot in the lobula (arrows), whereas flies whose glia express only Dmtn^1^ did not (M); neither did flies expressing wild-type Dementin in both neurons and glia (N). *dmtn^1^* flies whose neurons express only Dmtn^1^ further developed vacuoles (E, enlarged in E′), whereas *dmtn^1^* flies expressing wild-type Dementin in either neurons only, or in both neurons and glia did not (F, G, quantified in K). (H) Western blot and quantification of Futsch/MAP1B in rescued flies 1 day after eclosion and 10 days later showing that *dmtn^1^* flies neurons express only Dmtn^1^, have an age-related decline in Futsch levels, see also Fig. S2E; error bars are the SEM. This was accompanied by an age-related increase in APPLf levels in *dmtn^1^* flies expressing wild-type Dementin in glia only but not in those expressing wild-type Dementin in neurons only (I and J). *dmtn^1^* flies whose neurons express only Dmtn^1^ (O) showed deterioration of synaptic staining whereas flies whose glia express only Dmtn^1^ did not (P), neither did flies expressing wild-type Dementin in both neurons and glia (Q). (R) *dmtn^1^* flies whose neurons express only Dmtn^1^ (n = 118) had shorter lifespans than those who glia express only Dmtn^1^ (n = 138), or with wild-type Dementin in both neurons and glia (n = 54). Images are representative immunostained paraffin sections from 10–20 rescued *dmtn^1^* flies in each group fixed on the day after eclosion indicated and stained as indicated.

We found mislocalized Futsch in the somal compartment of subsets of medulla neurons in young *dmtn^1^* flies with expression of wild-type Dementin in glia only, but not those with expression of wild-type Dementin in neurons only or with expression in both neurons and glia (Figs. 7A to D), which had a staining pattern similar to that of wild-type flies (Fig. S3). By day 10, *dmtn^1^* flies whose neurons express only Dmtn^1^ showed reduced staining for Futsch (rescued in glia, Fig. 7E), whereas those whose glia express only Dmtn^1^ (rescued in neurons, Fig. 7F), or with expression of wild-type Dementin in both neurons and glia (Fig. 7G) retained the normal staining pattern. Quantification of Futsch levels, as detected by western blots using antibody 22C10, showed that this variation in staining was associated with age-related changes in relative levels of Futsch; in this assay, each lane was loaded with the equivalent of 4–6 heads from extracts prepared from separate pools of 18–42 flies in each group. Relative to the amounts detected in rescued 1-day old *dmtn^1^* flies whose glia express only Dmtn^1^ (rescued in neurons), Futsch levels in rescued *dmtn^1^* flies whose neurons express only Dmtn^1^ (rescued in glia) were nominally increased 1.5-fold at day 1, and were 0.8-fold at day 10 (Fig. 7H and Fig. S2F), statistical analysis of the intensities of the bands representing Futsch normalized to internal controls using ANOVA followed by Tukey's multiple comparison test showed that Futsch levels were significantly decreased in flies whose neurons express only Dmtn^1^ (rescued in glia) on day 10 compared to day 1 (p<0.05), consistent with a deterioration in the staining pattern for Futsch (Fig. 7E). Quantification of the levels of the ∼50 kDa APPLf, as detected by western blot using antibody dR-14, showed that it was differentially affected by expression of wild-type Dementin in either glia or neurons of *dmtn^1^* flies, and by age. In duplicate experiments, flies whose neurons express only *dmtn^1^* (rescued in glia) had 2.5 to 2.7-fold more APPLf at day 10 compared to day 1 (Fig. 7I), whereas the levels of APPLf in *dmtn^1^* flies rescued by expression of wild-type Dementin in neurons were 0.7 to 0.8-fold less on day 10 than day 1 (Fig. 7J). Each lane in Figs. 7I and J was loaded with the equivalent of 4 heads prepared from independent pools containing 22 to 42 heads each. Rescued *dmtn^1^* flies whose neurons express only Dmtn^1^ further showed evidence of neurodegeneration in the form of vacuoles (rescued in glia, Fig. 7E and E′, arrows) that were rarely found in any rescued 1 day old flies, in rescued 10 day old *dmtn^1^* flies whose glia express only Dmtn^1^ (rescued in neurons, Fig. 7F), or 30 day old flies with wild-type Dementin in both neurons and glia (Fig. 7G, quantified in Fig. 7K). Thus the age-dependent decrease in Futsch levels in glial-rescued *dmtn^1^* flies, whose neurons express only Dmtn^1^, may be caused by reduced synthesis in stressed neurons.

We further found patchy staining for Bruchpilot in the lobula complex of young *dmtn^1^* flies whose neurons express only Dmtn^1^ (rescued in glia, Fig. 7L), but not those whose glia express only Dmtn^1^ (rescued in neurons, Fig. 7M) or with wild-type Dementin in both neurons and glia (Fig. 7N), as detected using antibody nc82. The staining intensity for Bruchpilot in the optic lobe neuropils of *dmtn^1^* flies whose neurons express only Dmtn^1^ deteriorated with age (rescued in glia, Fig. 7O), but the staining for Bruchpilot in others did not (Figs. 7P and Q).

The above neuropathological features were associated with a severely reduced lifespan in *dmtn^1^* flies expressing wild-type Dementin in glia only compared to those expressing wild-type Dementin in neurons only, or in both neurons and glia (Fig. 7P).

Thus, while expression of wild-type Dementin in either the glia or neurons of *dmtn^1^* flies is sufficient to rescue developmental lethality, a lack of wild-type Dementin in the neurons of *dmtn^1^* flies lead to disruption to normal APPL metabolism, synaptic pathology, mislocalization of the microtubule-binding protein Futsch, neurodegeneration and early death.

Consistent with the reduced effect of neuron-specific RNAi for Dementin in development shown above, possibly due to a reduced efficacy of RNAi in neurons, 10-and 30-day old flies with RNAi for Dementin specifically driven by *elav-Gal4* in neurons showed staining for both Bruchpilot and Futsch similar to that obtained in wild-type flies, and normal lifespans (Fig. S3).

## Discussion

We previously found that human APP formed a complex with TMCC2, and that TMCC2 can both bind apoE *in vitro* and interact with it in cells [16]. Here we have used an independent, genetic, approach to show that human APP also interacts with the *Drosophila* orthologue of TMCC2, Dementin, and that Dementin interacts genetically with the *Drosophila* orthologue of APP, APPL. We therefore propose that the interaction between the Dementin and APP protein families is evolutionarily conserved. The study of Dementin-like proteins may therefore provide insights not only into the normal and pathological function(s) of APP-like proteins, but also of apoE.

We showed that mutation of *dementin* can cause neurodegeneration. The neurodegeneration in flies whose neurons express only *dmtn^1^* shares several pathological features with AD. Namely, disrupted metabolism of an APP-like protein, synaptic pathology, and disrupted distribution of Futsch, a protein with a close functional relationship to tau [26], [27].

Our finding that *dmtn^1^* flies rescued by neuron-specific expression of wild-type Dementin did not have neurodegeneration showed that in adult *dmtn^1^* flies the neurodegeneration was caused by disruption of Dementin function specifically in neurons, instead of incomplete rescue in glia or elsewhere. Since in adult flies APPL expression is primarily neuronal [28], this is supported by the observation that the levels of the *dmtn^1^*-induced APPLf did not increase over time in flies expressing wild-type Dementin in neurons, but did in those expressing wild-type dementin only in glia (i.e. in those with neurodegeneration).

Notably, *dmtn^1^* flies are missing only a small portion of the N-terminus of Dementin, revealing an important role for this portion of the molecule, and possibly accounting for the ease with which *dmtn^1^* flies were rescued. These findings also demonstrate the occasional utility of using hypomorphic alleles, as flies bearing null *dementin* alleles rescued by expression of Dementin specifically in neurons would not have developed into adults due to a lack of glial Dementin. The N-terminus of this protein family is specific to each paralogue, and almost completely conserved between homologues in the human and mouse genomes (Fig. S1). Thus the orthologue-specific N-terminus of Dementin-like proteins likely possesses a regulatory function, and the *dmtn^1^* allele produced a protein with dysregulated activity. As apoE can regulate the interaction between APP and TMCC2 in cultured cells [16], this suggests that modification of TMCC2 activity by apoE could contribute both to modified metabolism of APP and to neurodegeneration.

It is now recognized that while expression of autosomal dominant forms of APP in mice leads to the overproduction of Aβ, amyloidopathy and to behavioral defects, it does not lead to frank neurodegeneration [29]. In contrast, neurodegeneration is reported for *Drosophila* expressing either human Aβ or its *Drosophila* orthologue [30], [31]. In *Drosophila*, as in mice, neuronal expression of human APP causes behavioral defects, as shown in *Drosophila* by a failure of wings to expand after eclosion; we rescued this phenotype in *Drosophila* by ectopic production of Dementin, and exacerbated it by ablation of Dementin expression. In contrast, either ablation or ectopic expression of Dementin improved the eclosion rates of these flies. These findings suggest that the wing expansion and lethality phenotypes, while both caused by expression of human APP, are the consequences of divergent underlying cellular phenomena. Divergent effects of APP expression are also suggested by the observation that although neuronal expression of human APP is semi-lethal to *Drosophila* during development, it is protective in several adult *Drosophila* models of neuronal stress [32], [33]. In the present study, mere presence of the APPLf was not sufficient to cause neurodegeneration in *dmtn^1^/+* flies, or those with RNAi for Dementin specifically in neurons. This may be due to a protective effect of wild-type dementin in *dmtn^1^/+* flies, or to residual amounts of Dementin in flies with RNAi for Dementin in neurons. Alternately, as flies with neurodegeneration showed an age-dependent increase in APPLf levels, the levels of APPLf in *dmtn^1^* heterozygotes, or flies with neuron-specific RNAi may be too low to cause neurodegeneration. Resolving these contrasting *in vivo* effects of APP, Aβ and their *Drosophila* orthologues during development and in adults, and the effects of Dementin on these, may therefore lead to better *Drosophila* models for the functions of APP-like proteins and a better understanding of the effects of APP and Aβ in AD.

We showed that mutation of Dementin could both disrupt APPL metabolism and cause neurodegeneration, however our flies differ from most APP-related models of AD in that they do not over-produce Aβ. The sequence of the transmembrane and juxtamembrane regions of APPL and APP, from which the Aβ peptide that accumulates as plaques in AD is derived, are dissimilar. Nevertheless, the age-dependent accumulation of APPLf in adult *dmtn^1^* flies with neurodegeneration may conceptually recapitulate accumulation of Aβ-like peptides and other fragments of APP as seen in AD. In mammals, a fragment similar to the APPLf we observed would likely be degraded to smaller fragments by the wider variety of proteases that cleave APP, and subsequently by γ-secretase, whereas only one endogenous *Drosophila* protease, Kuzbanian, is recognized to constitutively cleave the juxtamembrane region of the APPL ectodomain in neurons. The apparent molecular weight of the APPLf observed in flies that either bear the *dmtn^1^* allele, or have neuron-specific RNAi for Dementin, suggests aberrant proteolysis within the ectodomain of APPL by unknown protease(s), rather than by α- or γ-secretase. This ∼50 kDa fragment of APPL likely originates through proteolysis or degradation of holoAPPL rather than sAPPL, as specifically modifying the metabolism of sAPPL would not be expected to alter the levels of holoAPPL, as observed. Since γ-secretase does not cleave transmembrane proteins with long extracellular domains, the ∼50 kDa APPLf therefore most likely contains the transmembrane and intracellular regions of APPL. However, the precise natures of the N- and C-terminal residues of the ∼50 kDa fragment of APPL we observed remain to be definitively established.

The mechanism by which Dementin deficiency leads to the production of APPLfs could nevertheless involve the non-proteolytic functions of presenilin previously described [7], [8]. Presenilin is strictly required for the production of Aβ from the C-terminal fragment of APP; our previous finding that TMCC2 and apoE together increased both total Aβ production and the ratio of Aβ42 to Aβ40 suggested that together apoE and TMCC2 stimulated an interaction between APP and γ-secretase. However, this interaction was evident only in the presence of both the “Swedish” mutation in APP, which increases production of the 99-amino acid C-terminus of APP that is a direct substrate for γ-secretase and of apoE, indicating a differential cellular interaction of TMCC2 not only with AD-risk versus normal forms of apoE, but also of APP [16]. The *Drosophila* orthologue of apoE is unknown, or may not exist, these experiments could therefore not be repeated in this study. Notably, however, a similar phenomenon to that which we observed for APPL in Dementin flies has been observed in presenilin 1-deficient mouse cells. In the absence of presenilin 1, short C-terminal fragments of APP accumulate due to a lack of γ-cleavage, as is now well established [7], [8]. However, for APP and the mouse APP paralogue, the APP-like protein 1, a ∼42 kDa C-terminal fragment accumulated in addition to shorter α-secretase cleaved fragments [34]. Further studies will be required to determine if Dementin can mediate an interaction between APPL and *Drosophila* presenilin.

In AD that has been confirmed *post-mortem*, neurodegeneration correlates better with tauopathy than with amyloidopathy. The impact of *dmtn^1^* on the cellular distribution of Futsch is potentially analogous to tau pathology as observed in AD, since it is established that in AD tau is mislocalized to the somatodendritic compartment [35]. We rescued Futsch mislocalization in *dmtn^1^* homozygotes by expressing wild-type Dementin in neurons without completely rescuing the effect of *dmtn^1^* on the production of APPLfs, and RNAi for Dementin specifically in neurons did not induce Futsch mislocalization. The effect of *dmtn^1^* on Futsch localization may therefore be independent of its effect on APPL; it may also be that loss of Dementin in neurons has less severe consequences than does expression of pathogenic forms, as has been shown in mice for other AD-related genes such as apoE and APP, and more recently for tau [36]. Alternately, since RNAi rarely completely suppresses protein production, a small amount of wild-type neuronal Dementin may be sufficient to protect against Futsch mislocalization in our flies.

Our study further demonstrated that the effects of Dementin expression are at least in part non-cell-autonomous. Since Dementin is not predicted to be a secreted protein, and the mammalian orthologue TMCC2 is located mostly within the endoplasmic reticulum [16], this may be due to downstream effects. The non-cell autonomous effect of Dementin expression was shown through the contrasting developmental effects of expressing wild-type Dementin in either the neurons or glia of *dmtn^1^* flies, and of RNAi for Dementin driven specifically in neurons or glia. The incomplete rescue of the optic lobe phenotype in *dmtn^1^* flies by expression of Dementin in glia could be accounted for by differences in the timing and/or pattern of *repo-Gal4* versus Dementin expression, and *repo-Gal4* may not be expressed in all glia [37]. However, this would not account for the complete rescue of development in *dmtn^1^* flies with wild-type Dementin expressed specifically in neurons. Thus, *dmtn^1^* retained at least partial activity in glia and affected brain development only in the absence of wild-type Dementin in neurons.

Development of the *Drosophila* inner optic lobe neuropils, the medulla and lobula complex, is poorly studied relative to that of the retina and lamina. To our knowledge, this study has provided the first evidence that glia are necessary for medulla development, as has been previously shown for the lamina and retina [38]. While lamina glia are required for development of both the lamina and the retina, and Dementin expression was detected in both neurons and glia of the medulla, it was detected in lamina neurons, but not in lamina glia (Fig. 2E), and only transiently in retinal neurons. A recent study has uncovered a number of “concentric” neural and glia-specific transcription factors involved in medulla development [39], which will aid future studies on the mechanisms underlying the role of Dementin in development of the inner optic lobe neuropils.

The importance of glial Dementin in development also establishes that Dementin has function(s) that extend beyond its interaction with APPL. Neither loss of APPL [28], expression of human APP [19], [20] or APPLsd [22] results in the neuroanatomical defects we observed in this study. The human and mouse genomes each contain four Dementin orthologues (Table 1), and data at the Allen Brain Bank (www.brain-map.org) show that three are expressed in the mouse brain. Thus, the differing glial and neuronal functions of *Drosophila* Dementin may be represented in the mammalian brain by different mammalian paralogues. While we can only speculate as to these other roles for Dementin-like genes in glia, our previous finding that apoE binds to the C-terminal region of TMCC2 [16], which is highly conserved in this family (Fig. S1), suggests that apoE might modify the biology of glia and other cell types through several members of this protein family, for example in inflammation, and in blood-brain barrier integrity [40].

## Materials and Methods

### Analysis of dementin-related sequences

Dementin-related sequences were collected and aligned based on homology using the ClustalW program [41] followed by manual refinement; the consensus sequence derived using seaview [42], and phylogenetic analysis was performed using the service at phylogeny.fr [43]. Coiled-coiled domains were predicted by the TMHMM service [44].

*Flies*— *UAS-RNAi* (*dementin*), line 37338, and *UAS-Dcr2* flies were obtained from the Vienna *Drosophila* RNAi Centre (Austria); this RNAi line is predicted to not have off-targets; all experiments using RNAi were performed in the presence of *UAS-Dcr2*. *dmtn^1^* flies are the *PBac{RB}(CG1021)[e01970]* line from the Exelixis collection at Harvard University (MA, USA); the reported insertion site was confirmed by inverse PCR. For glial-rescued flies, the *dmtn^1^* allele was recombined with *repo-Gal4*. *dementin-Gal4* (*P{GawB}NP6590*) was supplied by the *Drosophila* Genome Resource Centre (Kyoto, Japan). *UAS-APP695* (*P{w[+mC] = UAS-APP695-N-myc}TW6*), and *P{w[+mC] = UAS-GFP.nls}8* flies which express GFP with a nuclear targeting signal were obtained from the Bloomington Stock Center (IN, USA). For MARCM, P{neoFRT}19A, P{tubP-GAL80}LL1, P{hsFLP}1, w[*]; P{UAS-mCD8::GFP.L}LL5 flies were crossed *with P{neoFRT}19A; +; P{GawB}NP6590* flies, these stocks were obtained from the Bloomington Stock Center (IN, USA). *UAS-dementin* flies were made by amplifying the cDNA sequence from clone RE27645 obtained from the Berkeley *Drosophila* Genome Project (CA, USA), and cloning into pUAST; the construct was confirmed by sequencing and injected into w^1118^ embryos at Rainbow Transgenic Flies, Inc. (CA, USA).

All flies were raised on cornmeal agar, and when phenotypes were to be analyzed, at a density of 40–50 larvae per 10 ml of food.

For the analysis of wholemount brains by immunofluorescence, larval or adults brains were dissected and fixed in 4% paraformaldehyde, 1.6% L-lysine in phosphate-buffered saline for 20–30 minutes, washed with 1% Triton X-100 in phosphate-buffered saline (PBX) and blocked with 10% normal goat serum in PBX. Images were captured using a Zeiss510meta confocal microscope.

For the analysis of paraffin-embedded sections, flies were fixed overnight in Carnoy fixative (60% ethanol, 30% chloroform, 10% acetic acid), processed through 3 2-h incubations in methylbenzoate at room temperature, followed by a 1∶1 mixture of methylbenzoate and paraffin at 65°C and three changes of paraffin at 65°C (45 min each). Analyses were performed on 7 µm sections.

### Antibodies

Antibody 385 raised against the Dementin-derived peptide QSANADVLGSERLQ was raised in rabbits at Eurogentec (Belgium), under Belgian and EU welfare regulations (Directive 86/609/EEC), and used at a concentration of 1∶1000 in 5% milk, 150 mM NaCl, 50 mM Tris.Cl pH 7.4; anti-APPL antibodies dR-14 and dC-12 targeted to separate epitopes within the C-terminal region of the APPL ectodomain (communication from Santa Cruz Biotechnology) were obtained from Santa Cruz Biotechnology and used at a concentration of 1∶100 in PBS 0.2% fish gelatin, 0.1% Triton X-100; APPL antigen recognition was best observed in fresh samples or when samples were stored at −80°C. Rabbit polyclonal antibody A5137 directed against the final 20 amino acids of human APP was a gift of C. Miller (Kings College, London) [45]. Antibodies mAb24b10 (anti-Chaoptin), 8D12 (anti-Repo), nc82 (anti-Bruchpilot), and 22C10 (anti-Futsch) were obtained from the Developmental Studies Hybridoma Bank developed under the auspices of the NICHD and maintained by The University of Iowa, Department of Biology, and used at concentrations of 1∶20, 1∶20, 1∶100 and 1∶50, respectively. For detection of bound antibody in wholemount preparations, Alexa 488-conjugated goat anti-mouse secondary antibody (Invitrogen) was used at 1∶500, for detection of primary antibody bound to paraffin sections, Alexa 543-conjugated goat anti-mouse secondary antibody (Invitrogen) was used at 1∶500. The anti-tubulin antibody used was clone DM1A from Sigma and used at 1/2000.

### Western blots

All blots shown were performed on samples separated using SDS-PAGE gels containing 10% acrylamide and transferred to nitrocellulose; western blots were routinely developed using the ECL system (Amersham).

### Quantitation of APPL

APPL levels were quantitated using ImageJ software. Following normalization to loading controls, the levels of each APPL-reactive band was expressed as relative to the total amount of APPL detected in isogenic *w^1118^* flies from the same western blot.

*Statistical analyses—* Statistical analyses performed were ANOVA followed by Tukey's multiple comparison test on GraphPad Prism.

## Supporting Information

Figure S1 **Alignment of sequences orthologous to Dementin.** Sequences were initially aligned using ClustalX, followed by manual refinement. A minimal consensus sequence for this protein family, using in addition members others than those shown, was derived using the seaview software [42], followed by manual refinement and is shown in bold. Dashes indicate poor conservation of sequence and distance, X's indicate conservation of distance but not sequence. The locations of predicted coiled-coil domains are indicated by a row of c's, and transmembrane domains by a row of t's, both placed over the aligned sequences. The epitope against which antibody 385 against Dementin was raised is shown underlined and in bold.
(DOCX)

Figure S2 **A. Suppression of Dementin expression in adult heads using RNAi driven by** ***tubulin-Gal4*** **, each lane was loaded with the equivalent of 3 heads prepared from pools containing 15 to 20 heads each. B and C.** Expression pattern of *dmtn-Gal4* in the adult brain. *dmtn-Gal4* was used to drive nuclear GFP. (B) horizontal section of optic lobe showing expression in glia and other cells of the lamina and medulla. (C) Vertical section of the central brain showing expression in ensheathing glia (eg) and other cells associated with the mushroom body peduncle (Mp), central complex (Cc), and other neuropils. La, lamina; Me, medulla; Lo, lobula complex. xg, chiasm glia; sg, surface glia; cg, cortex glia. D. Western blots showing APPLf in the heads of flies with RNAi driven specifically in neurons or bearing the dementin alleles e01970 or f07621. E. Replicates of experiment described in Fig. 4B and 4D showing that *dmtn^1^* flies produce a ∼50 kDa fragment of APPL, each lane was loaded with the equivalent of 6 heads prepared from independent pools containing 18 to 42 heads each; APPL was detected by western blot using antibody dR-14. F. Replicates of experiment described in 7H showing the impact of rescuing *dmtn^1^* flies by expressing wild-type Dementin in either neurons or glia on the levels of Futsch on the days post-eclosion (PE) indicated; each lane was loaded with the equivalent of 4 heads prepared from independent pools containing 22 to 42 heads each, Futsch was detected with antibody 22C10. l.c., loading control (tubulin). G. Two replicates of experiment described in Fig. 3B, showing no significant impact of ectopic expression of Dementin or RNAi for Dementin in neurons on human APP levels. Extracts were prepared from the heads of flies expressing human APP, or which also have either RNAi for Dementin, or ectopic expression of Dementin. Each lane was loaded with the equivalent of 3 to 4 heads prepared from pools of 15 to 25 heads each.
(TIF)

Figure S3 **Neuronal RNAi for Dementin does not significantly alter staining for Futsch or Bruchpilot, or shorten lifespan.** (A–D) Sections from flies with RNAi targeted to Dementin were prepared on the indicated days after eclosion and stained as indicated. Images are representative of those obtained for 7 to 12 flies in each group. (E) Neuronal RNAi does not significantly alter lifespan; flies are *Dcr2/elav-Gal4; +; UAS-RNAi/+* (n = 117) or *Dcr2/+;+;UAS-RNAi/+* (n = 52). la, lamina; me, medulla; lo, lobula complex. (F to I) Paraffin sections of optic lobes of prepared from wild-type flies fixed on the indicated days after eclosion and stained for Bruchpilot or Futsch as indicated.
(TIF)

Figure S4 **Single optical sections of the optic lobe of third-instar larvae expressing nuclear-localized GFP (green) under control of** ***dmtn-Gal4*** **and stained either with antibody mAb24b10 (magenta), which detects chaoptin in the retinal axons, S4A, or with antibody 8D12 (magenta), which detects repo in glial nuclei, S4B.** In S4B, the nuclei of cells expressing both GFP and Repo appear white, . Confocal images were processed using ImageJ.
(TIF)

Movie S1 **Animated sections produced from stacked optical sections of the optic lobe of a third-instar larva expressing nuclear-localized GFP (green) under control of** ***dmtn-Gal4*** **and stained with mAb24b10 which detects chaoptin in the retinal axons (magenta) as shown in Fig. S4A.** Images were processed using ImageJ.
(AVI)

Movie S2 **Animated sections produced from stacked optical sections of the optic lobe of a third-instar larva expressing nuclear-localized GFP (green) under control of** ***dmtn-Gal4*** **and stained with antibody 8D12 (magenta), which detects repo in glial nuclei, the nuclei of cells expressing both GFP and Repo appear white, as shown in S4B.** Images were processed using ImageJ.
(AVI)
