# Supplementary material for: Neurodegeneration in a Drosophila Model for the Function of TMCC2, an Amyloid Protein Precursor-Interacting and Apolipoprotein E-Binding Protein
Source: PLoS One. 2013 Feb 7;8(2):e55810. doi: 10.1371/journal.pone.0055810 (PMC3567013; doi:10.1371/journal.pone.0055810)
Supplement: Figure S1 — Alignment of sequences orthologous to Dementin. Sequences were initially aligned using ClustalX, followed by manual refinement. A minimal consensus sequence for this protein family, using in addition members others than those shown, was derived using the seaview software [42], followed by manual refinement and is shown in bold. Dashes indicate poor conservation of sequence and distance, X's indicate conservation of distance but not sequence. The locations of predicted coiled-coil domains are indicated by a row of c's, and transmembrane domains by a row of t's, both placed over the aligned sequences. The epitope against which antibody 385 against Dementin was raised is shown underlined and in bold. (DOCX) [file pone.0055810.s001.docx]

Figure S1. Alignment of sequences orthologous to Dementin. A minimal consensus sequence for this protein family was derived using seaview software^50^, followed by manual refinement and is shown in bold. Dashes indicate poor conservation of sequence and distance, X’s indicate conservation of distance but not sequence. The locations of predicted coiled-coil domains are indicated by a row of c’s, and transmembrane domains by a row of t’s, both placed over the aligned sequences. The epitope against which antibody 385 against Dementin was raised is shown underlined and in bold.

Hs.477547 --------------------------------MEPSGSEQLFEDPDPGGKSQDAEARKQTESEQKLSKMTHNALENINVIGQGLKHLFQHQRRRSSV

Mm.425352 --------------------------------MEPSGSEQLYEDPDPGGKSQDAEARRQTESEQKLSKMTHNALENINVIGQGLKHLFQHQRRRSSV

Mm.273785 (TMCC2) MKRCKSDELQQQQGEEDGAGMEDAACLLPGADLRHGEASSANSAGGPTSDAGAAVAPNPGPRSKPPDLKKIQQLSEGSMFGHGLKHLFHSRRRSRER

Hs.6360 (TMCC2) MKRCRSDELQQQQGEEDGAGLEDAASHLPGADLRPGETTGANSAGGPTSDAGAAAAPNPGPRSKPPDLKKIQQLSEGSMFGHGLKHLFHSRRRSRER

Mm.23047 -------------------------------------------------------------------------------------------------

Hs.370410 -------------------------------------------------------------------------------------------------

Cin.32211 ---------------------------------------MEQVDVDDIEEHDGEVIVDAAEPINREHKSSFLPKSVLQLTKFNRAKVADTSSTTSTS

Dm.4403 (Dementin)---------------------------------------MRHNSPVSRERASEAAAATQTAAATAGGATAHSAGGTAGGSAAATTTAGGATSGSGTA

Hs.672606 (Tex28) -------------------------------------------------------------------------------------------------

Mm.475616 (Tex28) -------------------------------------------------------------------------------------------------

Cel.23608 -------------------------------------------------------------------------------------------------

**Consensus -------------------------------------------------------------------------------------------------**

Hs.477547 SPHDVQQIQADP--------------EPEMDLESQNACAEIDGVPTHPTALNRVLQQIRVPPKMKRGTSLH--------------SRRGKPEAPKGS

Mm.425352 SPHDVQQIQTDP--------------EPEVDLDSQNACAEIDGVSTHPTALNRVLQQIRVPPKMKRGTSLH--------------SRRGKSEAPKGS

Mm.273785 (TMCC2) EHQASQEAQQQQQQQGLSDQDSPDEKERSPEMHRVSYAVSLHDLPARPTAFNRVLQQIRSRPSIKRGASLHSS--GGSGG---RRAKSSSLEPQRGS

Hs.6360 (TMCC2) EHQTSQDSQQHQQQQGMSDHDSPDEKERSPEMHRVSYAMSLHDLPARPTAFNRVLQQIRSRPSIKRGASLHSSSGGGSSGSSSRRTKSSSLEPQRGS

Mm.23047 -------------------------------------------MPGSDTALT---------------------------------------------

Hs.370410 -------------------------------------------MPGSDTALT---------------------------------------------

Cin.32211 APQTPTDGDVTN--------------PMDDSHATLGVEKEINSVVRSRSLENYYREQLDRNDHKKSFKARLFG--------------STTALPTISA

Dm.4403 (Dementin)SANTNSNSSASS---------------STVAAAQAAVYSGGNTVTGSLGSAGVVARGFRSHSPTHRRRSRERQ--------------RRTHGSDQGG

Hs.672606 (Tex28) -------------------------------------------------------------------------------------------------

Mm.475616 (Tex28) -------------------------------------------------------------------------------------------------

Cel.23608 -------------------------------------------------------------------------------------------------

**Consensus -------------------------------------------------------------------------------------------------**

Hs.477547 PQINRKSGQEMTAVMQSGRPRSSSTTDAPTSSAMMEIACAAAAAAAACLPGEEGTAERIERLEVSSLAQTS---SAVASSTDGSIHTDSVDGTPDPQ

Mm.425352 PQINRKSGQEVAAVIQSGRPRSSSTTDAPTSSSVMEIACAAG----VCVPGEEATAERIERLEVSSLAQTS---SAVASSTDGSIHTESVDGIPDPQ

Mm.273785 (TMCC2) PHLLRKAPQDSSLAAILHQHQGRP----RSSSTTDTALLLADGSSAYLLAEEAESIG--DKGDKGDLVALSLPSGPGHGDSDGPISLDVPDGAPDPQ

Hs.6360 (TMCC2) PHLLRKAPQDSSLAAILHQHQCRP----RSSSTTDTALLLADGSNVYLLAEEAEGIG--DKVDKGDLVALSL--PAGHGDTDGPISLDVPDGAPDPQ

Mm.23047 --VDRTYSD-------PGR--------------------HHRCKSRVDRH---------------------------GSDTN--LNFDVPDGILDFH

Hs.370410 --VDRTYSD-------PGR--------------------HHRCKSRVERH--------------------------GGSDTNL--NFDVPDGILDFH

Cin.32211 PSFLRRRNVHDECDEGGGLKLNKRKQRMKGDVTADVEFMWQPSTTREPVHQARSSMDS--TNEIQPIVAPL--LMTTASDVSY-DAVDGSPGVSEAQ

Dm.4403 (Dementin)LLAYSGLVGVNDMTDFLGPQQGGG-----GGGGGGGGGGGGGGSAGTGS----------GLEDSRLSGNEDYYSSFVSDEFDSSK--KVHRRCHERS

Hs.672606 (Tex28) --------------------------------------MVLKAEHT---------------------------------LSSSEDGPSGPSSLADGG

Mm.475616 (Tex28) --------------------------------------MVLKVEST--------------------------------LSSFCEDCPSSHTSFSDGE

Cel.23608 ---------------------------------------------------------------------------------------KSSEGTCSSI

**Consensus -------------------------------------------------------------------------------------------------**

cccccccccccccccccccccccccccccccccccccccccccccccccccccccc

Hs.477547 QQKILKLTEQIKIAQTARDDNVAEYLKLANSA--------DKQQAARIKQRTKAAIAHLVFEKKNQKSAQTILQLQKKLEHYHRKLREVEQNG-IPR

Mm.425352 QQKILKLTEQIKIAQTARDDNVAEYLKLANSA--------DKQQAARIKQRTKAAIAHLVFEKKNQKSAQTILQLQKKLEHYHRKLREVEQNG-IPR

Mm.273785 (TMCC2) HQKILKITEQIKIEQEARDDNVAEYLKLANNA--------DKQQVSRIKQRTKAAIEHLVFEKKNQKSAQTIAQLHKKLEHYRRRLKEIEQNG-PSR

Hs.6360 (TMCC2) HQKILKITEQIKIEQEARDDNVAEYLKLANNA--------DKQQVSRIKQRTKAAIDHLVFEKKNQKSAQTIAQLHKKLEHYRRRLKEIEQNG-PSR

Mm.23047 RQKILKVTEQIKIEQTSRDGNVAEYLKLVSSA--------DKQQAGRIKQKVKLNADSLVFEKKNQKSAHSIAQLQKKLEQYHRKLREIEQNG-VTR

Hs.370410 KQKILKVTEQIKIEQTSRDGNVAEYLKLVNNA--------DKQQAGRIKQKVKLTADSLVFEKKNQKSAHSIAQLQKKLEQYHRKLREIEQNG-ASR

Cin.32211 QQKILKISEAIKLEQSTRDENVGDYLKLAGNA--------DKQQVARIKSRNRQLVEHIVFEKKNQKSNAAIAQLKKKFDTYHRRLREIESSGAVGR

Dm.4403 (Dementin)NTKIQCTKESIRQEQTARDDNVNEYLKLAASA--------DKQQLQRIKASSVQAIDRLVFEKKNQKSAHNISQLQKKLDNYTKRAKDLQNHQFQTK

Hs.672606 (Tex28) RHRILYLSEQLRVEKASRDGNTVSYLKLVSKA--------DRHQVPHIQQLAHNLQDSVAFEKVNQRASATIAQIEHRLHQCHQQLQELEEG-----

Mm.475616 (Tex28) KHRIFYLSEQLRVEKASRDENTMSYLKLVSKA--------DRHQAPHIRKLARNVREGVAFERVNQRTSATIAHIERKLYQCHQQLKELEEG-----

Cel.23608 VQKLIEIKDKLRALNEKREADVEKFLSITRQSEISRGVGADNPQRARIRN GDEREKAKNFERQNRKHAHETEMLQKKLIDYEERLKLVD-------I

**Consensus XQKILKLTEQIKIEQAXRDDNVAEYLKLANNA**--------**DKQQXXRIKQRXKXXXXXLVFEKKNQKSAQXIIQLQKKLEXYHRKLEEXEQNG**----

ccc

Hs.477547 QPKDVF----RDMHQGLKDVGAK-VTG----FSEGVVDSVKGGFSSFSQATHSAAGAVVSKPREIASLIRNKFG-SADNIPNLKDSLEEGQVDDAG-

Mm.425352 QPKDVFR----DMHQGLKDVGA-KVTG----FSEGVVDSVKGGFSSFSQATHSAAGAVVSKPREIASLIRNKFG-SADNIPNLKDSLEEGQVDDG-G

Mm.273785 (TMCC2) QPKDVLR----DMQQGLKDVGAN-MRAGISGFGGGVVEGVKGSLSGLSQATHT---AVVSKPREFASLIRNKFG-SADNIAHLKDPMEDGPPEEA-A

Hs.6360 (TMCC2) QPKDVL----RDMQQGLKDVGAN-VRAGISGFGGGVVEGVKGSLSGLSQATHT---AVVSKPREFASLIRNKFG-SADNIAHLKDPLEDGPPEEAA-

Mm.23047 SSKDISKDSLKEIHHSLKDAHV-KSRTAPHC-----LESSKSSMPGVSLTPPVFV---FNKSREFANLIRNKFG-SADNIAHLKNSLEEFRPEASP-

Hs.370410 SSKDISKDHLKDIHRSLKDAHV-KSRTAPHC-----MESSKSGMPGVSLTPPVFV---FNKSREFANLIRNKFG-SADNIAHLKNSLEEFRPEASA-

Cin.32211 QSKSL------------RDVGAN-LRD----FSGGVVDSVKGGLSGLQQATQNAAGAIASKPKDLASKLKNKFG-SADNLSSLK--YEEGGLED---

Dm.4403 (Dementin)SQHRQPREVLRDVGQGLRNVGGN----------------IRDGITGFSG-------SVMSKPREFAHLIKNKFG-SADNINQMSEAELQGM**QSANAD**

Hs.672606 (Tex28) -------------------------------------------------------------------------------------------------

Mm.475616 (Tex28) -------------------------------------------------------------------------------------------------

Cel.23608 -------------------------------SGEYEPSPTKSRVFPTGIRKAKGM-TETMVNAPIEFAQRVKSAFSADNVNSTQNGTTGAPKTGQST

**Consensus -PKDVRR-----DMQGLKDVGAN**----------------**VKGGSTGXXXXXXXXXXXAVSKPREFAXLIRNKFG-SADNIXXLKSGLEEXXXEX**---

Hs.477547 -----------------------------------------------------------------------------KALGVISNFQSSPKYGSEED

Mm.425352 -----------------------------------------------------------------------------KALGVISNFQSSPKYGSEED

Mm.273785 (TMCC2) -----------------------------------------------------------------------------RALSGSATLVSSPKYGSDDE

Hs.6360 (TMCC2) -----------------------------------------------------------------------------RALSGSATLVSSPKYGSDDE

Mm.23047 -----------------------------------------------------------------------------RAYGGSATIVNKPKYGSDDE

Hs.370410 -----------------------------------------------------------------------------RAYGGSATIVNKPKYGSDDE

Cin.32211 ----------------------------------------------------------------------------------TSLDY---RYTSADD

Dm.4403 (Dementin)**VLGSERLQ**QVPGAGTSTGSGGGGQN--NNTGGAGSGTG--------------------------------------------------KFNSDNGSE

Hs.672606 (Tex28) -CRPEGLLLMAESDPANCEPPSEKALLSEPPEPGGEDG-PVNLPHASRPFILESRFQSLQQGTCLETEDVAQQQNL---------------------

Mm.475616 (Tex28) -CSPTSSVLKVGSGLDSHKQPSGKVSYSKLSKPGGEDSLPINVARSS---TLESHLSEMQQRKFSDKKYVAQQQKLL--------------------

Cel.23608 FFTTRKSADTDEVESNAVHKNRGAKRNSSTLPPNLSLTSPDPLSDSSDPESRPGSAADETSNVPYHTADNSLYLPPNHPYHSAHAAPS---------

**Consensus -----------------------------------------------------------------------------KALXXXXXXXXXPKYGSDDE**

cccccccccccccccccccccccccccccccccccccccccccccccccccccc

Hs.477547 CSSATSGSVGANSTTGGIAVGASSSKTNTLDMQSS----GFDALLHEIQEIRETQARLEESFETLKEHYQRDYSLIMQTLQEERYRCERLEEQLNDL

Mm.425352 CSSATSGSVGANSTTGGIAVGASSSKTNTLDMQSSG----FDALLHEVQEIRETQARLEDSFETLKEHYQRDYSLIMQTLQEERYRCERLEEQLNDL

Mm.273785 (TMCC2) CSSASASSAGAGSNSGAGPGGALGSPRSNTLYGAPG---NLDTLLEELREIKEGQSHLEDSMEDLKTQLQRDYTYMTQCLQEERYRYERLEEQLNDL

Hs.6360 (TMCC2) CSSASASSAGAGSNSGAGPGGALGSPKSNALYGAPG---NLDALLEELREIKEGQSHLEDSMEDLKTQLQRDYTYMTQCLQEERYRYERLEEQLNDL

Mm.23047 CSSGTSGSADSNGNQSFGAGGTSTLDSQG----------KIAKIMEELREIKVTQTQLAEDIEALKVQFKREYGFISQTLQEERYRYERLEDQLHDL

Hs.370410 CSSGTSGSADSNGNQSFGAGGASTLDSQG----------KLAVILEELREIKDTQAQLAEDIEALKVQFKREYGFISQTLQEERYRYERLEDQLHDL

Cin.32211 VSS-TSSIDIGLSISGPDSPHSGLRRVDPQYMQAIIN--QVAGVKSDLVSTQVAHQQFEVEWEDWKRLEQNTIDLLTRSLQEERFRCERLEVQLNDL

Dm.4403 (Dementin)CSSVTSESIPGGSGKSQSGASQYHI--------------VLKTLLTELAERKAENEKLKERIERLET-GQKEFNNLTATLESERYRAEGLEEQINDL

Hs.672606 (Tex28) ---------------------------------------LLQKVKAELEEAKRFHISLQESYHSLKERSLTDLQLLLESLQEEKCRQALMEEQVNGR

Mm.475616 (Tex28) ----------------------------------------LQKMKEELTEAKKVHASFQVSHQSLKESHMIDVQRILESLQEKKTKQSLMEKQVNDH

Cel.23608 -------------------------------------EEGFNAIHEHLNSILQHLMLIDRKYDRLEDDIKKEIKFYAEALEEERFKTTKLEEILNEA

**Consensus CSSXTSGSA**-------------------------------**LXXLLEEIXEIKXXXXXLEEIQLELKQRYQRDYXLIXXTLQEERYRXERLEEQLNDL**

cccccccccccccccccccccccc ttttttttttttttttttt

Hs.477547 TELHQNEILNLKQELASMEEKIAYQSYERARDIQEALEACQTRISK-MELQQQQQQVVQLEGLENATARNLLGKLINILLAVMAVLLVFVSTVANCV

Mm.425352 TELHQNEILNLKQELASMEEKIAYQSYERARDIQEALEACQTRISK-MELQQQQQQVVQLEGLENATARNLLGKLINILLAVMAVLLVFVSTVANCV

Mm.273785 (TMCC2) TELHQNEMTNLKQELASMEEKVAYQSYERARDIQEAVESCLTRVTK-LELQQQQQQVVQLEGVENANARALLGKFINVILALMAVLLVFVSTIANFI

Hs.6360 (TMCC2) TELHQNEMTNLKQELASMEEKVAYQSYERARDIQEAVESCLTRVTK-LELQQQQQQVVQLEGVENANARALLGKFINVILALMAVLLVFVSTIANFI

Mm.23047 TELHQHETANLKQELASAEEKVAYQAYERSRDIQEALESCQTRISK-LELHQQEQQTLQTDAVN---AKVLLGKCINVVLAFMTVILVCVSTLAKFV

Hs.370410 TDLHQHETANLKQELASIEEKVAYQAYERSRDIQEALESCQTRISK-LELHQQEQQALQTDTVN---AKVLLGRCINVILAFMTVILVCVSTIAKFV

Cin.32211 TELHQREVTNLKQELFSMEEKVEYHASERARDMQEAIESCQTRLAK-MELQQQQ--LVSVDGLENATARALLGKLINLLLSVMAVLLVLVSTVSGLL

Dm.4403 (Dementin)TELHQNEIENLKQTIADMEEKVQYQSDERLRDVNEVLENCQTRISK-MEHMSQQQ-YVTVEGIDNSNARALVVKLINVVLTILQVVLLLVATAAGII

Hs.672606 (Tex28) LQGQLNEIYNLKHNLACSEERMAYLSYERAKEIWEITETFKSRISKLEMLQQVTQ--LEAAEHLQSRPPQMLFKFLSPRLSLATVLLVFVSTLCACP

Mm.475616 (Tex28) LQRYLDEICHLKQHLACTEEKMAYLSYERAKEIWDVMEIFKSRITKLETLQQATQ--LEMMASLRTRPKDFLFRFISLLLTLTTILLVVVSTLCSCP

Cel.23608 VELQQAEIATLKEQ-NLMATRVDYQHNDRFRNVEENMESLQNHLVR---IENALMDVRQVKLTSNVWQRVALNAG-NIVVELLKIALFVVASILDLV

**Consensus TELHQNEXXNLKQELASMEEKVAYQSYERARDIQEALEXCQTRXSKXXELQQQQQQQVQLEGXXXNAARXLLGKIXNXXXXXLAMVLVVSTXXXXXX**

ttttttttttttttt

Hs.477547 VPLMKTRNRTFSTLFLVVFIAFLWKHWDALFSYVERFFSSPR---------------

Mm.425352 VPLMKTRNRTFSTLFLVAFIAFLWKHWDALFSYVDRLFSPPR---------------

Mm.273785 (TMCC2) TPLMKTRLRITSTALLLLVLFLLWKHWAS-LTYLLEHVLLPS---------------

Hs.6360 (TMCC2) TPLMKTRLRITSTTLLVLVLFLLWKHWDS-LTYLLEHVLLPS---------------

Mm.23047 SPMMKSRSHILGTFFAVTLLAIFCKNWDHILCAIERII-IPR---------------

Hs.370410 SPMMKSRCHILGTFFAVTLLAIFCKNWDHILCAIERMI-IPR---------------

Cin.32211 KPLTKSPVRVISTVVVIISIIIAYKTWDTIP-MMSSMM-SSR---------------

Dm.4403 (Dementin)MPFLKTRVRVLTTFLSICFVIFVIRQWPDVQDIGSGLVRHLKQSLVVK---------

Hs.672606 (Tex28) SSLISSRLCTCTMLMLIGLGVLAWQRWRAIPATDWQEWVPSRCRLYSKDSGPPADGP

Mm.475616 (Tex28) LPLLSSRLRIFIVFMIIGLGTLAWQKRHVISIIDWQAWVPFKWRQDLKDAKPPSDGH

Cel.23608 RPLTGSRNR-SAMAFGLVFL-----AIFFGHHLQKVTYLFGGS--------------

**Consensus XPLMKTRXRXXXXXXXXXXXTVLWKXWDXXXXXXXXXXXXPK**---------------
