# Supplementary figures and images for: Neurodegeneration in a Drosophila Model for the Function of TMCC2, an Amyloid Protein Precursor-Interacting and Apolipoprotein E-Binding Protein
Source: PLoS One. 2013 Feb 7;8(2):e55810. doi: 10.1371/journal.pone.0055810 (PMC3567013; doi:10.1371/journal.pone.0055810)

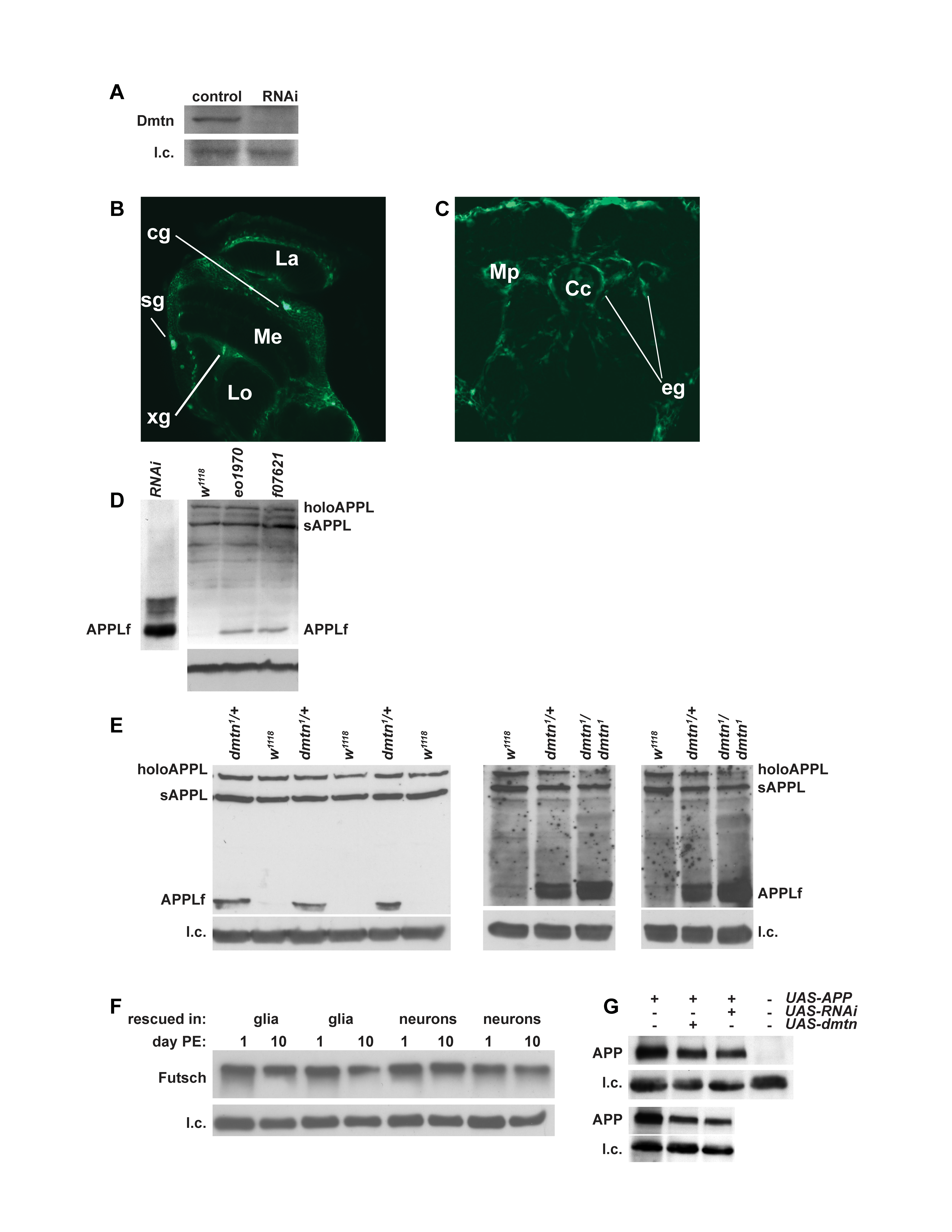

Supplement: Figure S2 — A. Suppression of Dementin expression in adult heads using RNAi driven by tubulin-Gal4 , each lane was loaded with the equivalent of 3 heads prepared from pools containing 15 to 20 heads each. B and C. Expression pattern of dmtn-Gal4 in the adult brain. dmtn-Gal4 was used to drive nuclear GFP. (B) horizontal section of optic lobe showing expression in glia and other cells of the lamina and medulla. (C) Vertical section of the central brain showing expression in ensheathing glia (eg) and other cells associated with the mushroom body peduncle (Mp), central complex (Cc), and other neuropils. La, lamina; Me, medulla; Lo, lobula complex. xg, chiasm glia; sg, surface glia; cg, cortex glia. D. Western blots showing APPLf in the heads of flies with RNAi driven specifically in neurons or bearing the dementin alleles e01970 or f07621. E. Replicates of experiment described in Fig. 4B and 4D showing that dmtn1 flies produce a ∼50 kDa fragment of APPL, each lane was loaded with the equivalent of 6 heads prepared from independent pools containing 18 to 42 heads each; APPL was detected by western blot using antibody dR-14. F. Replicates of experiment described in 7H showing the impact of rescuing dmtn1 flies by expressing wild-type Dementin in either neurons or glia on the levels of Futsch on the days post-eclosion (PE) indicated; each lane was loaded with the equivalent of 4 heads prepared from independent pools containing 22 to 42 heads each, Futsch was detected with antibody 22C10. l.c., loading control (tubulin). G. Two replicates of experiment described in Fig. 3B, showing no significant impact of ectopic expression of Dementin or RNAi for Dementin in neurons on human APP levels. Extracts were prepared from the heads of flies expressing human APP, or which also have either RNAi for Dementin, or ectopic expression of Dementin. Each lane was loaded with the equivalent of 3 to 4 heads prepared from pools of 15 to 25 heads each. (TIF) [file pone.0055810.s002.tif]

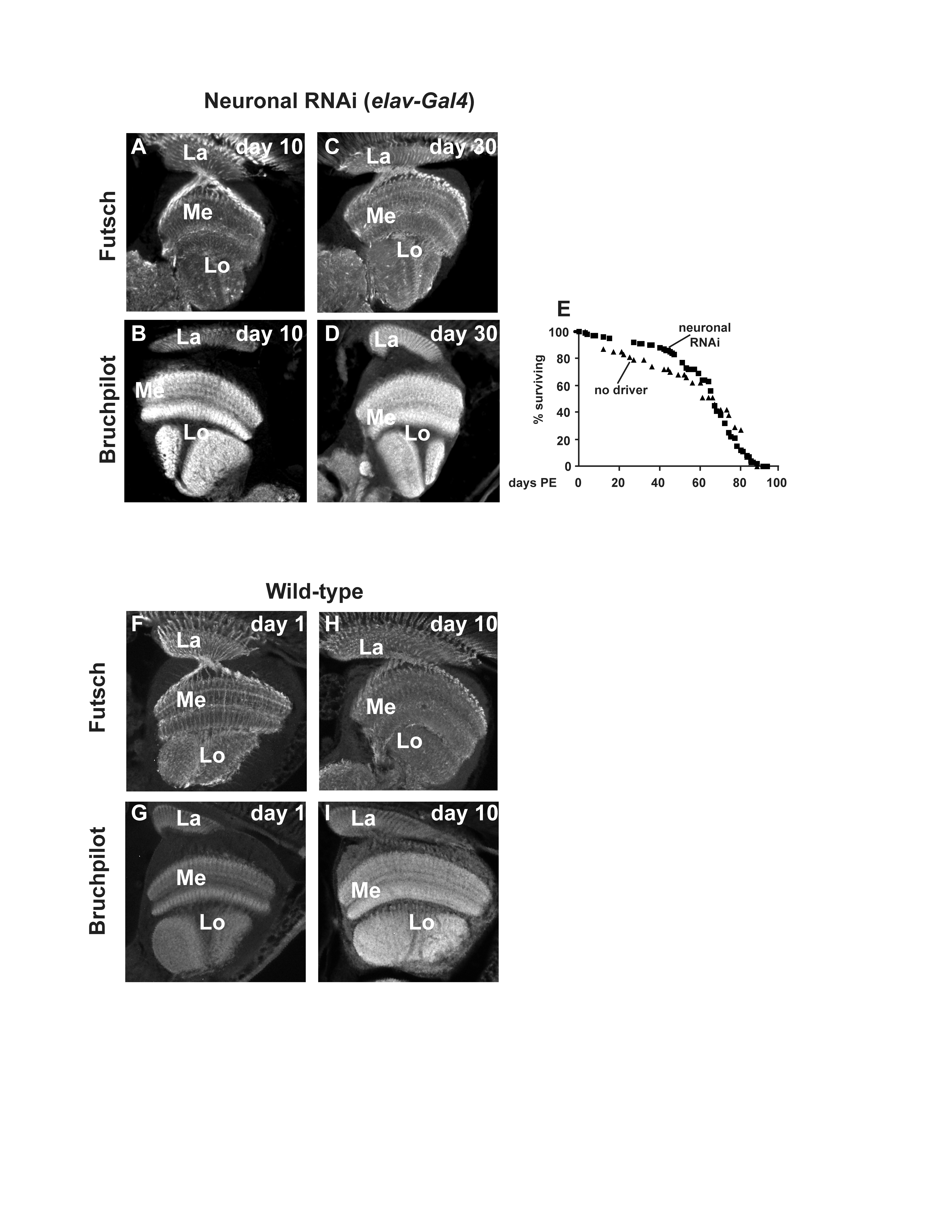

Supplement: Figure S3 — Neuronal RNAi for Dementin does not significantly alter staining for Futsch or Bruchpilot, or shorten lifespan. (A–D) Sections from flies with RNAi targeted to Dementin were prepared on the indicated days after eclosion and stained as indicated. Images are representative of those obtained for 7 to 12 flies in each group. (E) Neuronal RNAi does not significantly alter lifespan; flies are Dcr2/elav-Gal4; +; UAS-RNAi/+ (n = 117) or Dcr2/+;+;UAS-RNAi/+ (n = 52). la, lamina; me, medulla; lo, lobula complex. (F to I) Paraffin sections of optic lobes of prepared from wild-type flies fixed on the indicated days after eclosion and stained for Bruchpilot or Futsch as indicated. (TIF) [file pone.0055810.s003.tif]

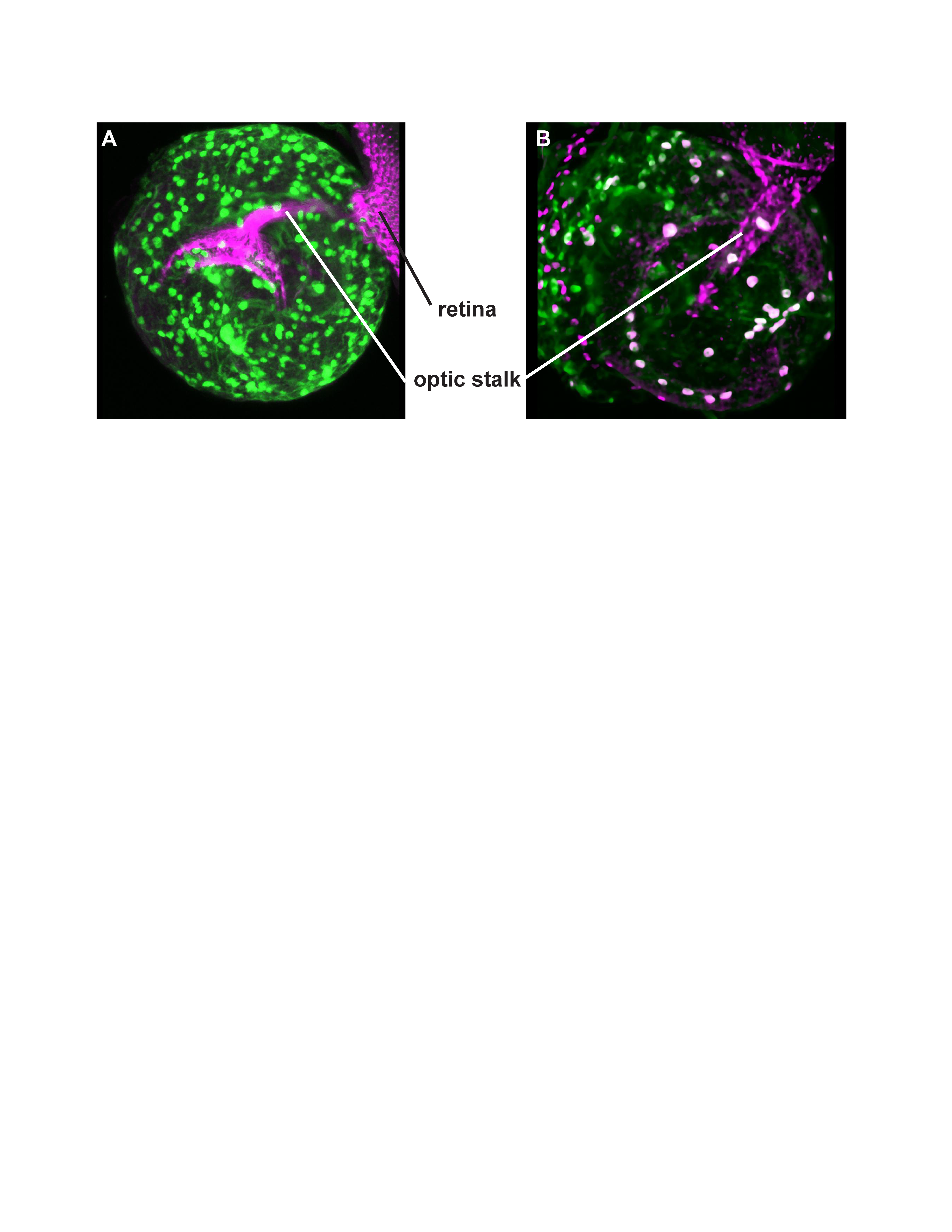

Supplement: Figure S4 — Single optical sections of the optic lobe of third-instar larvae expressing nuclear-localized GFP (green) under control of dmtn-Gal4 and stained either with antibody mAb24b10 (magenta), which detects chaoptin in the retinal axons, S4A, or with antibody 8D12 (magenta), which detects repo in glial nuclei, S4B. In S4B, the nuclei of cells expressing both GFP and Repo appear white, . Confocal images were processed using ImageJ. (TIF) [file pone.0055810.s004.tif]
